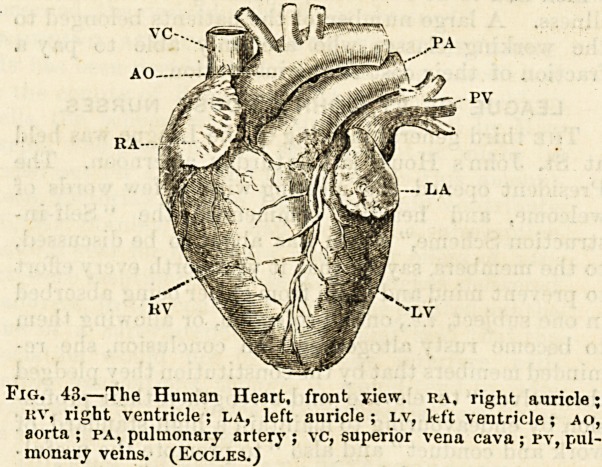# The Hospital. Nursing Section

**Published:** 1902-05-17

**Authors:** 


### The Hospital.

### Nursing Section.

Contributions for this Section of "THE HOSPITAL" should be addressed to the EDITOR, "THE HOSPITAL" NURSING SECTION, 28 & 29 Southampton Street, Strand, London, W.C.

No. 816.—VOL. XXXII. *SATURDAY, MAY 17, 1902.*

### Notes on News from the Nursing World.

### NURSING THE VICTIMS OF THE VOLCANO.

THE fact that wherever there is sickness and death, the nurse goes hand in hand with the doctor is emphasised afresh by two announcements following the news of the awful disaster in the West Indies. At the English colony of St. Vincent, which has been affected in a minor degree, there were nurses on the spot, but they are necessarily only a limited number, and it is no doubt correctly stated that they are overworked. Nor has any time been lost in dispatching nurses to the island of Martinique. With praiseworthy promptitude the Barbados Government at once sent three trained nurses to the scene of the catastrophe, and they landed at the ruined capital on Tuesday.

### "FROM A NURSE'S POINT OF VIEW."

WE think it desirable that, in addition to high officials, one of the rank and file of nurses should reply at some length to Miss M. F. Johnston's attack. The article in another column is by a nurse whose experience justifies her claim to a hearing. It will be observed that she lightly passes over the charges made against the profession to which she belongs. The value of her remarks lies chiefly in her vindication of the hospital authorities who have been held up to reprobation. Against the fiction of irresponsible writers she sets the facts of her own life in one of the principal institutions, and she shows how far removed from actuality is the picture given of unfeeling matrons and careless sisters. She does not claim that hospital work is easy, but she insists that it is not drudgery. In language that will go home to the hearts of many, she describes the manner in which the nurse is treated in time of sickness; and altogether it may be said that her reply, all the more forcible because of its simplicity, completes the refutation of an indictment which had been brought against the nursing profession.

### FEVER NURSES FOR FEVER CASES.

THE matron of a well-known trained nurses' institute has had a painful and, we trust, unusual experience. A doctor sent to her for an ordinary nurse, and she despatched one who had happened not to have had, or even nursed, scarlet fever. The nurse got to her case in the evening, and the next morning found that the patient had a rash on her body. The case was in the country, where there was no post out that day, and the nurse thought as she had been so long in contact with the fever she had better stay. The consequence has been serious, for the nurse was taken ill, and people have been unpleasant to the matron. Such a case shows how urgently desirable it is that in applying for a nurse for an infectious case information should be given as to the nature of the disease.

### A PERSONAL QUESTION.

THE correspondence started last month by "Inexperience" as to sleeping in a patient's room is brought to a close to-day. The remarkable interest excited is attested not merely by the letters we have published, but also by the very large number which we have necessarily been compelled to exclude. At the outset we remarked that for a female nurse to sleep in the room of a private male patient can only be regarded as allowable under very exceptionable circumstances, and to this we have nothing to add. In our issue of April 26th, however, "Medicus," in an admirable letter, which evidently represents the feelings of many nurses, enlarged the controversy, and, while affirming that the practice of a nurse sleeping in a male patient's room is "highly undesirable," proceeded to more severely condemn that of permitting a woman to pass a catheter on a man. Nor, in spite of some protests which have been raised, can there be any doubt that the condemnation is deserved. Yet here again it is impossible to lay down a hard-and-fast rule. A nurse who possesses every attribute of modesty may not consider it an outrage on her feelings to be asked, in the event of an emergency, to undertake such cases, and she is not to be blamed if she consents. But it is quite certain that there should be no compulsion in the matter. Moreover, it must be borne in mind that nurses are not all of the same age, and that what may be right for some may at least have the appearance of being very improper for others. We hope that the controversy, which we have permitted to go on somewhat beyond the usual time, will really prove of practical value and will invest young nurses with sufficient courage to refuse to discharge duties which they feel they cannot perform without a sense of impropriety.

### CARELESSNESS AT BROMSGROVE.

ANOTHER instance of the want of care manifested at some of the provincial infectious hospitals is afforded at Bromsgrove by the report of a committee of inquiry which was appointed in view of the fact that some of the patients had been dosed with marking-ink in mistake for beef-tea. It appears that one nurse had put the ink into a beef extract bottle, and that another had administered it; and the committee very properly came to the conclusion that both were to blame. They also blamed the matron who, it transpired, was cognisant of the circumstance that the beef extract bottle had been used for ink for the past nine months, and she, as well as the nurse who put the ink into the bottle, has been asked to resign. The nurse who administered the dose of ink anticipated enforced resignation by leaving. It may be hoped that the next matron will at least see that bottles are used for the purpose for which they were intended and are correctly labelled.

### A CURIOUS MISCONCEPTION.

A CORRESPONDENT, who sends neither name nor address but a marked copy of a religious paper containing an attack on the Roman Catholic Church, protests against our "continual advocacy of the employment of Romanist nurses in hospitals." We only notice this statement because it is so utterly at variance with facts. Neither the employment of Roman Catholic nor of Protestant nurses has ever been advocated by THE HOSPITAL. Our correspondent misunderstands the position. We deprecate the exclusion of thoroughly qualified trained nurses from hospitals or infirmaries either on the ground that they are Protestants or Roman Catholic. The question of their religion is one that concerns themselves, and neither hospital authorities nor Boards of Guardians are justified in raising it. We are glad to acknowledge that as a rule they refrain from doing so. It becomes our duty when exceptions are brought under our notice to condemn the practice.

### CHELTENHAM AND THE WOMEN'S MEMORIAL TO QUEEN VICTORIA.

THE sum of £297 2s. 6d. has been sent to the Jubilee Nursing Institute in London as the contribution of the people of Cheltenham to the Women's Memorial to Queen Victoria. This is all the more creditable to Cheltenham because there is a great desire on the part of the inhabitants to render financial assistance to the Victoria Nursing Home in the town, and a certain number of persons refused to give to a fund which was not to benefit it. To ignore national claims because there are local needs is not patriotic, nor in the long run is the Victoria Home likely to suffer. We have no doubt that those who have contributed to the larger object will now do their share in supporting the smaller.

### A REACTIONARY PROPOSAL AT HEXHAM.

A SUGGESTION was made at a meeting of the Hexham Board of Guardians that as both a nurse at the infirmary and a workhouse porter are wanted, it would be desirable to obtain the services of a married couple who would respectively undertake the work required. It is fair to add that the Hexham Guardians did not go to the length of assuming that the wife of a porter would be a trained nurse, but some of them maintained that it was not necessary that the nurse should be trained. Ultimately the, question was referred to the Workhouse Visiting Committee, who, it may be hoped, will report against the adoption of an utterly retrograde policy, which appears to have been advocated merely because a nurse recently appointed was not able to take up her duties. It cannot be impossible to obtain a qualified person in her place; but to put the untrained wife of a porter into the position would permanently discredit the Hexham Infirmary.

### EAST LONDON NURSES AT ST. PAUL'S CATHEDRAL.

ON Friday morning last week a special service was held at St. Paul's Cathedral for the members of the East London Nursing Society. The service, which consisted of morning prayer and celebration of the Holy Communion with an address, was in the north-west chapel. A good many nurses were present and some few communicated. The address was delivered by the Rev. R. W. Harris, Rector of St. George's-in-the-East, who said that although many of the alleged miracles at Lourdes and other places, and those worked by the Peculiar People, could not, in his opinion, be disproved, yet these were exceptions, and the beneficent work of healing, to which the life of Christ was so largely devoted, was now being carried on by physicians, surgeons, and nurses. He therefore appealed to the nurses to look upon their work as a continuation, so to speak, of Christ's work. The East London Nursing Society was undenominational in the best sense; no attempt to proselytise or persuade was attempted by the nurses; but at the same time he thought that it would be very hard if, when face to face with a dying man or woman longing for comfort and peace, they were not to speak a word of encouragement. There were, he added, people who would not have them do even this.

### THE NEEDS OF MIDDLESBROUGH.

THERE are several pleasing features in the annual report of the Middlesbrough Nursing Association for last year which we have just received. The Boards of Guardians increased their subscription from fifty guineas to £100; a lecture delivered on behalf of the Samaritan Fund realised £14 3s. 3d.; several special gifts of money are mentioned, including £2 2s. from the Workmen's Accident Fund at one of the works; the Imperial Tramway Company gave free passes to the district nurses, and the Middlesbrough Co-operative Society presented them with a handsome writing table for their sitting-room. The financial position, though not yet satisfactory, since there is still a balance due to the bank, has improved; and the work done by the four district nurses shows no relaxation of energy, the number of cases nursed being 688, and of visits paid 17,842. The value of the movement may be gathered from a paragraph in the report, which says, "On one of the coldest days of the winter babies were born in a home where there was neither food, fire, nor clothing for mother or children, and it was easy to see what the consequences of such destitution would have been if there had been no District Nursing Association to provide for their necessities." The lady superintendent thinks that if the cases attended "could be made widely known" the income of the society would be very largely increased. We agree with her; but cannot some of its enthusiastic supporters take care that, so far as Middlesbrough is concerned, the desired publicity is obtained?

### A NURSING ASSOCIATION FOR SOMERSETSHIRE.

IT has been decided to form a nursing association for the county of Somerset. At a meeting held in Wells, the scheme was explained in detail by Miss Joseph, who observed that she thought it might be carried out for £400, and that towards this amount Queen Victoria's Jubilee Institute would contribute £50, Mr. Stanley, M.P., had promised a subscription of £10 a year for five years, and other offers of support were mentioned. Miss Amy Hughes delivered an address, in the course of which she pointed out the value of a superintendent nurse, and said that the association would not in any way interfere with existing associations, though it would tend to raise the standard of the nurses. An influential committee was subsequently formed.

### QUEEN'S NURSES AT PETERBOROUGH.

THE annual report of the superintendent of the Peterborough District Nursing Association is satisfactory enough, and shows that the staff last year attended 161 cases, and paid nearly 5,000 visits. Tables are appended giving the nature of the cases attended, the final results, and the whereabouts of the cases. The report of the treasurer is not so satisfactory. The balance at the bank has been reduced from £39 to under £5, and the treasurer laments the "great deficiency in the contributions, owing to a want of collectors in some parts of the town." We notice that very many of the subscriptions are small, which justifies the conclusion that the class most concerned are not unresponsive. But some of the collectors have certainly been far less successful than others, and in this direction there must be room for an increase of energy.

### A DEFICIENCY AT LONGTON.

At the annual meeting of the Longton Sick Nursing and Samaritan Association a report of a character not creditable to the workers in the Potteries was submitted, since it shows that the expenses of the Association exceed the income by nearly £40, and that subscriptions and donations have fallen off. Of the work done by the nurses there is no complaint. One of them nursed in the year 134 cases and paid 3,929 visits; the other nursed 164 cases and paid 3,397 visits; and both are described as very attentive and devoted to their duties. It is hoped that the coffers of the organisation may be replenished by a ping-pong tournament on its behalf which was held in the Town Hal on Tuesday. But the men employed in the trade of the town who receive good wages, and whose families derive benefit from the services of the nurses, are the people to look to for an increase of income.

### THE MARY WARDELL CONVALESCENT HOME.

Accompanying the annual report of the Mary Wardell Convalescent Home for Scarlet Fever is a pathetic appeal which can scarcely fail to meet with a response. Miss Wardell needlessly apologises for intruding personal requests on the attention of the subscribers. The reminder that she is just 70 years of age, and that her sight is failing, instead of being resented, should prompt subscribers to send in their contributions before the end of the year, when "days are dark and short." Full particulars are given in the report of the work done, and of the difficulties which had to be overcome, including several cases of illness. A large number of the patients belonged to the working classes, who are only able to pay a fraction of their cost to the institution.

### LEAGUE OF ST. JOHN'S HOUSE NURSES.

The third general meeting of the League was held at St. John's House last Saturday afternoon. The President opened the meeting with a few words of welcome, and heartily commended the "Self-instruction Scheme," which was about to be discussed, to the members, saying that it was worth every effort to prevent mind and brain from either being absorbed in one subject, *i.e.*, one's profession, or allowing them to become rusty altogether. In conclusion, she reminded members that by the constitution they pledged themselves "to elevate and strengthen their profession by endeavouring to maintain a high standard of work and conduct" and also "to promote the usefulness and honour of the nursing profession." As there was a full agenda, the meeting was long and animated: it was followed by a social gathering, with tea and some good music contributed by various friends.

### "A STITCH IN TIME SAVES NINE."

There is always a danger of "the willing horse being worked to death," and nursing associations cannot be too careful not to overtax the physical strength of their staff, especially when the whole of the duties devolve upon one person. At the annual meeting of the Sutton-in-Ashfield Association it was stated that the nurse had attended 177 cases and paid 5,186 visits, that "the continuous work of the earlier period of the past twelve months had told upon her," and that the committee had granted her an extended rest. It appears, however, that for some time before the rest was given the nurse had been "far from well." In such circumstances it is much better to afford immediate relief. A nurse who is "far from well" pursues her avocation under disadvantages to her patients as well as to her own detriment.

### A LEFT-HANDED COMPLIMENT.

A woman residing in the East End of London had been attended for some time by one of the district nurses. At last, however, the doctor decided that it would be necessary for her to enter the hospital in order to undergo an operation. The nurse arranged to assist the patient to dress, and when she arrived she found the clergyman of the parish, who having heard of the impending change had called to wish his parishioner "God speed." "Well, nurse," said he, "I am glad to see you have come to give a helping hand to Mrs. N. You have attended her now for so many months, she will feel quite strange with different nurses." "Yes, indeed," the woman chimed in; "you don't know, sir, how good she has been to me. I don't know what I should have done without her. She is a real 'fallen angel,' she is!"

### DESCRIBED AS A "NURSE."

There is no evidence to show that Sarah Russell, who was sentenced last week to seven years' penal servitute for having caused the death of a married woman by an illegal operation, had received the training of a nurse, though she described herself as one. The "nefarious and mischievous trade" which Mr. Justice Ridley has done his best by the punishment inflicted on Russell to put down, is, of course, regarded with the utmost detestation by nurses, and it is a pity that inquiries were not made at the trial with the view of ascertaining how far the convict had a claim to belong to an honourable profession.

### SHORT ITEMS.

Nursing Sisters D. Pryde and E. P. Gibb, both A.N.S.R., arrived at Southampton from South Africa on the 9th inst.; both require two months' leave and return to South Africa.—Nursing Sisters F. Baker, V. D. Chawner, H. O. Luckie, who arrived in the *Montrose* on Monday, require ten days' leave before returning to South Africa; E. L. Collins requires one month's leave and returns to South Africa. Nursing Sisters M. Wilson and Monk-Mason, invalided home, have been granted three months' leave. —The contribution of the women of the Punjab to Lady Curzon's fund for the training of native midwives as a memorial to Queen Victoria amounted to over 65,700 rupees (£4,250).—Miss Chinnery, who was trained at Middlesex Hospital, and was for eight years sister in the Cama Hospital, Bombay, has been offered the appointment of lady superintendent of Walker Hospital, Simla.

### Lectures to Nurses on Anatomy.

By W. JOHNSON SMITH, F.R.C.S., Principal Medical Officer, Seamen's Hospital, Greenwich.

### LECTURE XVII.—THE HEART AND BLOOD-VESSELS.

THE heart is situated in the thoracic cavity lying behind the lower two-thirds of the breast-bone but projecting much further into the left than into the right side of the chest. It is a cone-shaped organ measuring in the adult about five inches in length and three and a half inches from side to side at its broadest part. The cone takes an oblique direction from above downwards and from right to left, the apex corresponding to a point on the surface of the chest about two inches below and one inch to the inner side of the left nipple in the male, and the base to a line drawn across the breast-bone a little below the well-marked trans-

FIG. 42.—Diagrammatic Section of Heart. A, aorta, semilunar valves at lower end; P, pulmonary artery; X, right auricle; Y, left auricle; R, right ventricle; L, left ventricle; D, septum between ventricles; E, apex of heart; VV, superior and inferior venæ cavæ; F, FI, pulmonary veins; Z, tricuspid; and ZI, mitral orifices; S, tricuspid; SI, mitral valves. The arrows show direction of the flow of blood, and (N) represents the flow to and from the lungs.

verse line between the second pair of ribs. If the finger be placed on the point corresponding to the apex, the "beats" of the heart may be distinctly felt.

Having noted in addition to these few dry but very useful details, that the heart is enclosed in a loose bag of fibrous tissue, which is called the *pericardium*, let us consider in the first place what work this important organ has to do, and secondly, how it is anatomically fitted to carry out such work.

The function of the heart is to propel a continuous stream of deteriorated and poisoned blood into the lungs, and when the blood has been purified in its progress through these organs, to again propel it in a continuous stream to all parts of the body. The heart has thus two circulations to keep up: a minor one, the *pulmonary circulation*, through the lungs; a major one, the *systemic circulation*, throughout the general system.

A clear idea as to how this is done may be gained by glancing over a diagram (fig. 42.). The diagrammatic heart here shown in a vertical section is a hollow organ containing four chambers, two, one above the other, on the right side, and two disposed in a like manner on the left side. The two chambers on each side communicate with each other, but there is no communication between the right and left sides of the heart, the partition wall, like that of two attached villas, being whole and intact from top to bottom. Each of these chambers, the walls of which consist of contractile and ever-acting muscle, is in direct communication with one or more blood-vessels, a single vessel opening into each of the two lower chambers, two into the upper chamber in the right side, and three or four into the corresponding chamber in the left side.

The two upper chambers, which are really ante-chambers and receiving-rooms, are called the *auricles*, the lower chambers, which discharge or pump out the blood are called the *ventricles*. The two vessels opening into the right auricle are two large veins—venæ cavæ—the single vessel communicating with the right ventricle is the *pulmonary artery*, the three or four vessels opening into the upper chamber or auricle on the left side are the *pulmonary veins*, and the single large and thickly-coated vessel passing from the left ventricle is the *aorta*. The partition wall between the right and the left chambers of the heart is called the *septum*.

The blood having traversed the body and become mixed with the products of the "wear and tear" of its different organs is discharged by the two venæ cavæ into the right auricle. It then passes into the right ventricle, the muscular walls of which pump it through the pulmonary artery into the lungs. After it has traversed these organs and disposed of its poisonous ingredients it is carried by the pulmonary veins into the left auricle, and finally after it has reached the left ventricle is forced by the very thick and strong muscular walls of this chamber into the aorta and all its arterial branches. The blood, after it has been thus distributed, is again collected by the veins, passing from venous branches to venous trunks and discharged into the *auricle* or ante-chamber on the right side, where we first met with it.

As the heart after each pumping action must receive into its chambers a further supply of blood, it follows that the

FIG. 43.—The Human Heart, front view. RA, right auricle; RV, right ventricle; LA, left auricle; LV, left ventricle; AO, aorta; PA, pulmonary artery; VC, superior vena cava; PV, pulmonary veins. (ECCLES.)

circulation cannot be kept up except by alternate movements of contraction and relaxation of the muscular walls of the ventricles and auricles. The order in which these motions are carried out was very clearly explained

### LECTURES TO NURSES ON ANATOMY.—*Continued.*

by Mr. G. H. Lewes: [1]—"The two ante-chambers (auricles) suddenly contract; immediately afterwards, but while the auricles are still contracted, the two chambers (ventricles) also contract, having been powerfully expanded by the rush of blood from the auricles. This contraction is named the *systole* of the heart. It continues for a brief moment, and is followed by a relaxation of the auricles, which is immediately succeeded by a relaxation of the two ventricles. This relaxation is named the *diastole.*

These associated movements are repeated from 70 to 75 times per minute in a healthy adult. The number of beats or pulsations each corresponding to a single succession of the above described motions varies in the two sexes and at different periods, and is much influenced by different conditions of both health and disease. It is higher in the female than in the male, and in the child than in the "grown-up" person. It is much increased in cases of high fever and acute inflammation and lower in exhausting disease and in conditions of collapse. Each beat can be felt an applying the fingers over the region of the heart, especially over its apex a little below and to the inner side of the left nipple, and by using a stethoscope or placing the ear directly to the wall of the chest, two sounds may be heard which, it is stated, may be imitated by pronouncing the words *lubd* and *dup*.

Having with the aid of our diagram gained some idea as to how the circulation of the blood is kept up by the unceasing rhythmical movement of the heart, the question may occur to some of us as to why the blood always moves in the same direction, and why, during the relaxation of first the auricle and then the ventricle, there is no reflux into both these chambers. Such reflux, which would seriously disturb the action of the heart, and which, indeed, causes the grave symptoms consequent on certain forms of heart disease is prevented when the organ is sound by a complex and elaborate arrangement of valves attached to the opening between the auricle and ventricle on both sides, and also to the openings of the pulmonary artery and the aorta. The valves attached to each of these two large blood-vessels consist in each case of three semilunar folds of membrane, which, during the contraction of the ventricles, are pressed back against the wall of the vessel, and, during the relaxation of these chambers, fall together so as to form a resistant circular septum or partition. From their shape these membranous folds both in the pulmonary artery and the aorta are called the *semilunar valves.*

The arrangement of the valves attached to the auriculo-ventricular orifices is not so simple. On the right side there is along the margin of the orifice a continuous zone of membrane, which zone, as it is followed away from its attachment becomes thinner and divides into three valves. Each of these valves is split up into a number of fringes to which are attached by means of distinct tendons several muscular columns the fixed ends of which are continuous with the muscular structure of the heart. There is a like arrangement in the left auriculo-ventricular orifice; but as this is smaller than the orifice on the right side, the valve also is smaller and presents two instead of three main divisions.

The membranous portion of this valvular arrangement with its three main divisions on the right side is called the *tricuspid valve*, and with its two divisions on the left side the *bicuspid* or *mitral valve.* The muscular columns are called the *columnæ carneæ*, and the small tendons by which these columns are attached to the fringed margins of the valves the *chordæ tendineæ.*

We shall now be able, I hope, to follow in a set of good plates or on a preserved specimen some of the more important of the anatomical details to which I have just now referred. On the anterior surface of the heart placed in its natural position (fig. 43), we shall see a transverse groove marking the boundary between the two ventricles below and the two auricles above, and also a more distinct groove running from base to apex which separates the right from the left side of the heart. The direction of this latter groove indicates that the right chambers occupy not only the right side but most of the front of the organ, whilst the two left chambers are placed behind as well as on the left side. If the cavities be laid open we shall see that the auricles have thin walls and smooth inner surfaces, whilst the ventricles have very thick muscular walls and their inner surfaces are rendered very uneven by interlacing bands and columns of muscle. We shall see also the expanded auriculo-ventricular valves with their fringed margins and fan-shaped tendinous attachments to the muscular columns of the ventricles, and be able to compare these with the much more regular and simple arrangement of the semilunar valves of the pulmonary artery and the aorta (fig. 42). We should also notice that behind each of these semilunar valves there is a distinct pouch or depression in the arterial well. In the bewildering jungle of blood-vessels attached to the base of the heart we should find no difficulty in distinguishing the pulmonary artery at the upper part and the front of the right ventricle, and above this pulmonary artery the large and conspicuous arch formed by the aorta (fig. 43). On each side of these two vessels may be seen a muscular pouch with irregular and dentated margins, which pouch is a part of the ante-chamber, and has imparted to the whole of this cavity its special name of auricle.

[1] The Physiology of Common Life.

### Beyond the Seas: Nursing in a Japanese Fever Hospital.

BY A TRAINED NURSE.

MY experiences in a Japanese fever hospital last year may be interesting to some of the readers of THE HOSPITAL. For some previous years I had been carrying on some medical work in connection with a mission in Tokio; I had learnt the language, and when my work there came to an end, I decided, before returning to England, to see, if possible, a little of the inner life of a Japanese hospital. I applied to the Government, and after various questions were asked me as to my reasons for wanting to enter, etc., I was, to my joy, accepted. I was asked to bring my own furniture, as they had none, so one bright day in April I sallied forth from my home with all things needful, and arrived at the hospital at tea-time. My room had nothing in it, the floor was covered with brown sacking, which the authorities thought I should like better than a polished one, and there was no privacy, as two sides of it were glass sliding doors.

### UNUSUAL EXPERIENCES.

After unpacking my boxes and drinking two or three cups of Japanese tea which the matron kindly brought me the house physician sent for me, and told me what wards he wanted me to look after, and what were my duties, and then he asked me to write down for a week what I would like for my meals, or if I could not do that to give him a cookery book, as the cook was not used to foreigners When that interview was over I was taken into the nurses' room, and introduced in a very novel way. We all sat down on the floor and as their names were called out the Japanese nurses all bowed very slowly, till their

### BEYOND THE SEAS.—*Continued.*

heads reached the floor, and I had to return the bow likewise. This was the finale; afterwards I retired to my room, supped, and went to bed. I had charge of a block of typhoid wards; each ward held four beds and had glass sliding doors, in front of which was a verandah running the length of the block, the wards opening off it. I was on duty generally from 7 A.M. to 6 P.M., with half an hour off for my mid-day meal. This does not mean that I worked hard all the afternoon; sometimes I had a good deal of leisure; but it signifies that I had to sit in my little room near the wards to superintend.

### Difficulties.

I had one or two difficulties. For instance, most of the nurses had never spoken to a foreigner before, and none of them could speak a word of English, so I had to give all my orders in Japanese. Then, though the treatment was very up-to-date, the nursing was bad. I do not want to convey the impression that all Japanese hospitals are alike, for I believe that in some the nursing is very good; but this was a fever hospital, and the great attraction good salaries to be got. Accordingly women who could neither read nor write were on the staff. Personally, I had nothing to do with the lowest class, as the authorities seemed to have picked me out the best. The nurses' hours were so long that if they did not work energetically you could not say much to them. One day they were on duty 17 hours, the next 10, the next 19, and so on all the summer months, because there were no night nurses, and the work had to be done between them.

### Curious Treatment.

The cases were generally very heavy, as it was usually a man, woman or child already suffering from kakke that had developed typhoid. These patients were never sponged nor put in a cold pack, whatever their temperatures might be. Their temperatures, pulses and respirations were taken every three hours, from 6 A.M. to 6 P.M., but in the night the patients were allowed to rest in peace. They were not fed unless with ice, neither were their temperatures, etc., taken, though of course when they were dangerously ill and needed it they had frequent hypodermic injections of camphorated oil night and day.

### Diets.

The patients, according to their condition, were put on one of four diets. First, for the very sick, rice water and milk; second, "Majiri"—rice water containing a small portion of rice and milk; third, "O kai," very soft rice with two eggs and milk; and, fourth, ordinary diet which was rice (cold or hot), vegetables, and occasionally fish. They were never fed except at their meal times—seven, noon, and evening, but those who were very ill were ordered weak wine and water frequently.

### Nurses' Salaries.

The salaries of the nurses were from 10 to 14 yen a month (in English money from £1 to 28s.), out of which they had to pay 3 yen a month for their food. They only had two days a month off duty, and were astonished at my wanting to go out every day. The nursing of delirious patients was especially difficult, for the nurses were so few that there were no specials, and the wards being so small you never knew what the patients were doing when your back was turned. No one seemed anxious when a delirious patient walked along the verandah, but a nurse only said: "Oh! it can't be helped," a speech the Japanese are very fond of, and assisted him back to bed.

### "O Soba Ya."

During my six months stay I tried to teach these girls how a typhoid patient ought to be nursed, but it seemed a hopeless task, as the majority stay such a short time, and then one had to begin the same thing over again with the next newcomer. However, in spite of my difficulties, I was very sorry when the day came for me to leave, for I had made many friends. I was not allowed to depart till I had been taken to a "O Soba Ya" and had a good feast of O Soba, eating it with chopsticks (O soba is a kind of food that looks like macaroni) and received a roll of lovely Japanese silk.

### "The Case against Hospital Nurses."

### A REPLY.—FROM A NURSE'S POINT OF VIEW.

Having read in the *Nineteenth Century and After* both Miss Johnston's article entitled the "Case against Hospital Nurses" and the two very excellent replies to it in this month's issue, I feel that perhaps a few words from one belonging to the rank and file of the nursing profession would not come amiss, though taken as a class nurses are more generally known as doers of deeds rather than as writers of words. I am a trained nurse of some years' standing and hold the certificate of one of the first hospitals not only in London but in Europe, a fact of which I am justly proud.

### Different Sorts of Nurses.

All nurses who have worked for any length of time in a hospital know that there are all kinds of nurses, even as there are all sorts and conditions of women, and that when a woman decides to take up the profession of nursing and to make it her life work, she certainly does not drop her nature as if it had been a loose-fitting mantle, and blossom forth into the ideal nurse all at once. Whatever shortcomings and faults she possesses go with her into the new life, and can only be overcome by daily striving and perseverance. That far more is expected, nay demanded, of a nurse than of any other woman in any walk of life is perhaps one of the highest compliments that can be paid to the profession, though that all will not possess the moral courage and strength to live up to those demands is evident. But shall that fact deter us from doing our utmost, even if while doing so we realise our many and grievous shortcomings?

### Privileges and Responsibilities.

The nurses of our time have many privileges that were altogether unknown to those of former years, but we must remember that just in proportion to these come greater and ever-increasing responsibilities; and if we accept the one, we must also not fail to grasp the latter. Every day there is something new to be learnt year in, year out, and it rests with each one of us individually to do our share and prove to the world at large that the life we have taken up is one of the best and demands from us all we have to give. If we recognise the possibilities, as well as the difficulties, of any responsible work, our convictions concerning the former will be of material help to us in overcoming the latter. The ideals we set before us, and endeavour to attain, will gradually become accomplished facts if the work is undertaken and carried out in that true spirit of perseverance which, no matter what the pitfalls may be which we fall into by the way, will always enable us to make a fresh start.

### The Life not Easy.

A nurse's life is not easy; no one is ever told that it is,

### "THE CASE AGAINST HOSPITAL NURSES."—*Continued.*

and yet there are never wanting people to come forward and offer themselves for training. That all do not succeed is not to be wondered at, seeing that they hail from every rank of society, many with a very wrong idea of what nursing is. It does consist of downright hard work, and in everyday language takes it out of one both mentally and physically; but it is as far removed from drudgery as light from darkness, and I think all who have attained to any degree of success in it will agree with me that it is, and always must be, a very soul-satisfying life, and that its fascinations grow as the "knowledge that is not learned of books" comes to those who devote themselves to the work of relieving human suffering.

The Aspersions on Hospital Authorities.

It is not, however, of ourselves as nurses that I desire particularly to write, nor do I even wish to attempt to vindicate ourselves before the public. I want especially to draw attention to the aspersions upon hospital authorities and officials in general. There may be a few training schools of a second or third rate order in existence where the welfare of the nurses is not considered, but these are most certainly in the minority. Surely the public do not believe that numbers of women, all of them come at least to years of discretion, would voluntarily remain in any institution where they would receive the treatment recorded in the "Case against Hospital Nurses" by Miss Johnston. The nurses are in every instance absolutely free agents. They are not asked to enter the hospital world. They do so entirely by their own choice, and if accepted as candidates, after being a certain length of time on trial, sign the agreement to remain for a given period of training of their own free will. How could anyone believe that they would do this if treated with so little consideration, in fact, as set forth with absolute cruelty. It is certainly not the salary that tempts them, for nurses are not as a rule well paid, and it is only those who secure the highest positions who can hope to save enough to retire upon and live in comfort when too old to continue work.

A Personal Experience.

Personally I am thoroughly acquainted with only one hospital. Perhaps I have been unusually fortunate in my experience, though I believe it is only that of hundreds of others who have received their training at one or other of the best acknowledged training schools.

In all truthfulness and sincerity, and without the slightest reservation on any one point, I can say with absolute certainty that from the day when, with several other new probationers, I entered this great hospital fresh from my sojourn of seven weeks at the preliminary training school, I have never had cause to regret my decision to become a nurse nor my choice of a hospital. As for the treatment I have received, it would be impossible to overrate the kindness and consideration bestowed upon me first and foremost by our matron, and to a greater or less degree by each of the hospital authorities. It is with much personal love and with all loyalty to her who has done so much for me and for each one of us, not only by her teaching and example, but by her whole life and by the faith she has in us, that I mention this, though on subjects about which one feels most deeply the least is often said.

The Nurse in Sickness.

And in sickness. There, too, I may claim the right to speak, though I am fully aware that by doing so I may be accused of possessing that failing so often attributed to nurses, viz., talking of their own health. I have been often "off duty" and in the sick-room with minor ailments. I have been really ill. I have also been very near death. Notwithstanding the extra work it causes when one member even of a large staff is often absent from her post, no hint was ever made of it to me, though naturally I was aware of the inconvenience it would mean, and every encouragement was always given me to get quite strong and well. When later I lay just on the borders of life and death, when all were uncertain what the issue would be, and hope still strove to triumph over what seemed absolute hopelessness, words would be altogether too inadequate and time too short to express all that was then done for my welfare. And my home people, what of them? They were summoned at the first moment when serious symptoms developed, and made welcome guests throughout that trying time. Always on looking back to the darkness of those days, when that awful struggle against death went on, the recollection of the true, loving human sympathy so freely given to them by all, stands out clear and undimmed. And with that remembrance still fresh in my mind I can never bear to remain silent when a word is spoken even indirectly against those to whom I owe so much. I shall always be proud to maintain with the forceful argument of personal experience that, let a member of the staff be ill, it matters not whether probationer, charge nurse, or sister, everyone instantly vies with each other as to how much they can do to help.

Fiction and Facts.

Finally, if Miss Johnston cares to take the trouble, then she, or anyone else who wishes to see the life going on in our great hospitals, should come unannounced and visit the wards. I think I may venture to say at all hospitals, but most certainly at this one, that large grim, smoke-begrimed, almost barrack-like place built right in the midst of the teeming multitudes in our sad East End, any day of the week, at any time, morning, noon (or night if they prefer it), they will be heartily welcome. Let them see everything, the hospital in general with the wards—accident, surgical, and medical—the theatres, the out-patient department, the receiving room, the kitchens, the laundry, the nurses' home with its sick-room, its dining-room, sitting-room, library, etc., where every comfort is provided, and I think when they have seen all and have heard from the patients' own lips what they think of the hospital—when they have seen the little children, those poor suffering scraps of humanity, put their arms round nurses' necks to show how much they love them, they will come to the conclusion that hospital officials and hospital nurses are not after all quite such inhuman people as they are sometimes painted.

And in conclusion let me say one word to those of my fellow-workers who, having borne the heat and burden of many a long day, read Miss Johnston's article. Discouraged and downhearted not a little that after all their efforts the public should have had put before them such a misrepresentation of facts, knowing that some at least of it will be believed, is it to be wondered at if they lose heart for a time? But let us all take fresh courage in both hands and start once more. Let the few grains of truth contained in that article spur us on to make greater efforts after that ideal which we all possess. Let us strive to raise the nursing standard ever nearer to perfection. Let us each do our best to walk worthy of the vocation which we have chosen.

### Wants and Workers.

District Nurse, Cawston Lodge, Haverland Park, thanks most sincerely the kind unknown friend for her parcel.

The Matron of the Home for Confirmed Invalids, Aubert Park, Highbury, would be grateful to anyone who could tell her where to obtain a second-hand propelling chair for a poor girl.

### Different Kinds of Enemata.

### EXAMINATION QUESTIONS FOR NURSES.

The question was as follows: "Give examples of the different kinds of enemata, and state the circumstances under which each kind is most frequently ordered by doctors."

### First Prize.

The different kinds of enemata are:—

*Purgative Enemata*, ordered for patients whose internal organs are not in a fit condition to allow of aperients being administered by the mouth, such as gastric or typhoid cases; after abdominal operations when it is necessary to keep the intestines quiet; in cases of obstruction and abdominal pain, when purging might be dangerous; when aperients have been given by mouth without success; before abdominal operation to supplement aperients in clearing the intestines; before vaginal or rectal examination; before the administration of an anæsthetic.

They may be soap and water injections, made by well mixing soft soap in water as warm as the patient can comfortably stand; in addition to this the doctor may order turpentine (usually about half an ounce), olive oil or castor oil. Oil injections, warmed olive oil, with or without the addition of soap and water; if oil only, from two to ten ounces may be given, and it may be retained for some hours, and followed by a soap and water enema. This is especially useful when the lower bowel is blocked with hard masses.

Turpentine enemata, with or without castor oil, sometimes ordered in thin gruel, sometimes in soap and water; this is often given for the relief of distension or flatus.

Glycerine warmed, or, better, mixed with a little hot water, often useful as an occasional alternative with soap and water, or before an emergency operation.

*Nutrient Enemata*, ordered when a patient is unable to take any or sufficient nourishment by the mouth; when the stomach or intestines are in need of absolute rest, as in gastric ulcer, operations on the stomach, intestines, etc, in cases of persistent vomiting; in mouth or throat cases when swallowing is impossible or undesirable in patients with disease of the œsophagus or stomach.

They usually contain milk and one or more of the following: Egg, beef tea, raw meat juice, brandy; doctors generally order them to be peptonised.

*Stimulant Enemata*, ordered in cases of collapse, sometimes after operation, heart failure, opium poisoning, and may be made of brandy and hot water, strong coffee, etc.

*Saline Enemata*, ordered in cases of collapse from operation or hæmorrhage, also for thirst in patients fed by enemata (one drachm of salt to each pint of water).

*Starch and Opium* or iced water enemata, ordered to check diarrhœa, the latter kind chiefly for infants; starch—from two to four ounces, the quantity of opium will be ordered.

*Medicated Enemata* for affections of the lower intestines as boracic injections for enteritis. "Robin."

### Second Prize.

*Simple Enemata.*—One ounce of soft soap to one pint of water (temperature 90°-100° Fahr.), inject one or two pints slowly. For constipation or before operations.

*Castor Oil.*—One ounce, or sulphate of magnesium, four ounces, may be added to the above.

*Olive Oil.*—Four ounces of warm olive oil. In cases of extreme constipation when there is a collection of hardened fæces in the rectum which cannot be removed by simple enemata.

*Glycerine.*—One to two drachms of warm glycerine for hardened fæces in the rectum.

*Turpentine.*—One ounce of turpentine to 12 ounces of thin starch or 1 pint of soap and water. In cases of flatulence after an abdominal operation.

*Starch and Opium.*—One ounce or two of thin starch (mixed first to a paste with cold water, then adding boiling water until thin enough), when nearly cold add 30 minims of tincture of opium. In cases of severe diarrhœa, hæmorrhage from the bowels (as in typhoid and malignant growth in the rectum).

*Salt.*—One tablespoonful of salt to one pint of warm water or thin gruel. Given to children in cases of thread worms.

*Nutrient.*—Four to six ounces of peptonised milk or beef tea, with stimulants if ordered by the doctor, or eggs beaten up in milk (temperature 90°-100° Fahr.). These are given in cases in which there is obstinate vomiting, or when it would be dangerous to give nourishment by the mouth, as in cases of gastric ulcer or after abdominal operations.

*Normal Saline.*—One drachm of salt to one pint of water (temperature 100° Fahr.). In cases of collapse after operations, also given to relieve thirst after abdominal operations.

*Medicines, e.g.*, chloral and bromide, etc., when there is obstinate vomiting.

*Ice* is sometimes given as iced water. Shamrock.

### The Character of this Month's Papers.

Taken as a whole the standard is not quite so good as last month. The competitors have been much too discursive and I have to complain of carelessness in not paying attention to our very few rules. Again and again candidates have been told to write their answers straightforwardly, without headings or marginal notes. Want of space entirely precludes the publishing of such answers; they are put on one side, whether bad or good, as debarred from consideration. A nurse who fails to conform to rules in a writing competition is more than likely to be slipshod in professional work, and to evade rules in more important matters. Very occasionally from pressure on space the rules are omitted; it is surely not too much trouble to glance back a month and make sure you have conformed to them. "Hopeful" sends no proper name or address. Alas! it is little use to be hopeful if one is unpractical.

### Difficulties in Administering a Soap and Water Enema Combined with Oil.

Only one nurse has noticed this difficulty, I should therefore like to speak of the best way to overcome it. Oil rises to the top of soap and water and remains there; the nozzle of the syringe being considerably below the surface, suction does not reach the oil till the vessel is emptied. Now it frequently happens that the patient's power of retention fails before all the liquid has been injected, consequently the bowel receives no oil. To avoid this, place the oil in a second small basin with only a couple of ounces of soap and water and inject it first, you can then pass to the larger basin and use as much as the circumstances of the case admit of. The same precaution should always be taken when medicines are to be administered in combination with ordinary or nutritive enemata. The medicine should be mixed with a small portion of it and administered first.

### Question for May.

What means would you employ to overcome the continual "slipping down" in bed of an absolutely helpless patient, who is ordered to remain in a semi-recumbent position, or is compelled to be sitting up in bed?

The Examiner.

### Rules.

The competition is open to all. Answers must not exceed 500 words, and be written on one side of the paper only. The pseudonym, as well as the proper name and address, must be written on the *same* paper, and *not on a separate sheet.* Papers may be sent in for fifteen days only from the day of the publication of the question. All illustrations strictly prohibited. Failure to comply with these rules will disqualify the candidate for competition. Prizes will be awarded for the two best answers. Papers to be sent to "The Editor," with "Examination" written on the left-hand corner of the envelope.

N.B.—The decision of the examiners is final, and no correspondence on the subject can be entertained.

In addition to two prizes honourable mention cards will be awarded to those who have sent in exceptionally good papers.

### A Character Sketch.

BY AN AUSTRALIAN NURSE.

She was a delicate-looking, white, and whining little woman, and I noticed, while helping her to bed, that she had the practised mannerisms of a regular invalid, that her clasp was feeble, and her voice subdued. She had looked so fragile and weak as she waited to be admitted by the ward door, that I had taken her bodily in my arms and carried her to her allotted bed.

"What do you complain of?" I inquired, pulling off her boots, which were very old, but lightly made, and still neat.

"Pain all over, and the doctor said a high temperature."

"The doctor doesn't generally tell the patient that," I said sharply.

She looked at me with wide innocent-looking eyes, then a smile dawned on her face.

"You don't understand," she said; "I'm a nurse."

"A nurse!"

"Trained at Mary's, five years ago, and have a certificate. Now I've come to this!" She looked down at her shabby garments in disgust, and involuntarily I asked "Why?"

"Got married," she summed up briefly; "and my little child is in your children's ward now."

The tears in her grey eyes checked my flow of questions; so I tucked her into bed, and gathered her things together, then reluctantly took away the screen, and she was "warded."

During the busiest part of the evening a gentle call from her attracted my attention. Flying to her side, I demanded what she wanted, pointing out to her how busy we were. My sharp tone evidently hurt her, for she did not answer, but turned her face into her pillow, and a sob shook the bed. Having no time to comfort her, I hurried off, feeling angry with myself, to finish the work which could not wait. After this I was particularly kind and attentive to this patient; in fact, her evident gentleness appealed to us all, and she was the envy of the other patients, who hated her. She used to lie quiet and uncomplaining, watching us with her big pathetic eyes, but she demanded a lot of fidgeting attention in her quiet way; hot-water bottles continually, drinks at odd times, and when we were ready to do things for her, or brought her meals she would be asleep, and seemed too exhausted to be disturbed She continually complained of great pain, for which she had morphia, and she always had a high temperature. Her one cry was for her baby, and she begged everyone to let her have a sight of him, though it was against the rules; so one day sister gave in and brought her the child and we thought the mother would break her heart when he was taken away again.

At last the physician's visiting day arrived. The house physician brought him up to the new patient saying she was a case which puzzled him greatly. The woman lay asleep as usual with her head buried in the bed-clothes. I thought they would never rouse her, but at last she slowly lifted her head, and looked at the doctor, her face even whiter than usual, and her eyes darker.

To our astonishment the doctor raised his eyebrows, and turning on his heel walked away. That evening the little woman was sent away from the hospital. "Hysteria; rubs the thermometer up; doctor knows her," was all the explanation we had of her rapid departure.

Two years afterwards, I was sent to help in a surgical ward.

"We want help," explained sister, "because of two big operations to-day, and there is a third patient who is very troublesome. She is continually crying out, and trying to get up, and disturbs the others dreadfully. She is in great pain and begs for morphia, which is restricted; they have made two incisions but find nothing, though she has a very high temperature."

Going to her bedside I immediately recognised the same little woman. "How is your baby?" I asked her.

"My baby?" She turned great innocent eyes to my face and stared in astonishment. "I haven't a baby? I'm not even married," she said.

"Why!" I cried, astonished in turn, "Don't you remember the child who was in here with you two years ago?"

"I've never been in here before," she asserted, so positively that it took my breath away. Then it dawned on me that she must be playing another big game, and thinking that a the nurses and doctors on the medical side were different to those on the surgical, she would not meet them. So far she had been safe. "Then you don't know me?" I queried.

"Never seen you before, and don't wish to again, for you are not at all soothing and the pain is cruel. I want morphia.'

Then she howled, till the patients on all sides looked terrified.

Sister called me and said: "That patient has not made a fuss before, with a nurse by her side, you must keep her quiet."

"Sister," I began, "she is an old humbug; was diagnosed two years ago as hysterical."

Sister looked angry. "How dare you," she said, "come to my ward and lay down the law about my patients? surely the doctor knows better than a probationer!"

Sorry that I had been wanting in tact in a strange ward, I went again to the originator of the trouble.

"Stop that noise and look at me, Mrs. Smith," I demanded.

"My name is Brown," she said sulkily, but looked, her eyes shirking mine.

"Look here," I continued, "it's no use playing upon me, for I know you! And you need not continue to make that noise; in fact, if you take my advice you would drop this farce altogether, and go home quietly. The incisions are almost healed, and if you stop shamming and rubbing up the thermometer, no one will be any the wiser."

However, she buried her head in the clothes, her old trick, and would not answer; so I started the round of temperature, taking hers first. Knowing her, I held the glass. After three minutes it was sub-normal, and I tried it again with the same result. After this she lay perfectly quiet.

"Did you give that patient morphia?" asked sister, as I passed her.

"No; her temperature was sub-normal when I took it, sister."

"Nonsense, nurse! Give me the glass."

She marched off and put it under the woman's arm, talking meanwhile to another patient; then she brought it back.

"How do you account for this, nurse? it is 104!"

"Hot-water bottles, or rubbing," I returned indignantly.

"You are incorrigible," she said. "If you had seen how bravely she took chloroform twice, you would not be so hard-hearted." Evidently sister had a soft corner for the delicate little woman, just as we had had. Nevertheless, a few days afterwards I heard that there had been a big row in that ward, and that Mrs. Brown had departed. I saw her again, when waiting on an out-patient doctor. When he had seen her, he looked up with a twinkle in his eyes and said "Mark that little woman, nurse, for she is the cleverest sham of her day, and has had no end out of the hospital. She has been an in-patient at every one in London at least once, and has had every conceivable disease and operation possible; yet she is alive still, and deceives and puzzles some of our cleverest men, when she gets a chance. I have a great respect for her myself; she is so clever and, so brave, and has such confidence in the medical profession."

### Everybody's Opinion.

[Correspondence on all subjects is invited, but we cannot in any way be responsible for the opinions expressed by our correspondents. No communication can be entertained if the name and address of the correspondent are not given as a guarantee of good faith, but not necessarily for publication. All correspondents should write on one side of the paper only.]

### A PROBATIONER'S GRIEVANCE.

"A Lover of Justice" writes: A probationer, when training at a poor law infirmary, had the misfortune to have a diseased hand after she had been in the building nearly a year. It was very slight for weeks, and even months. It went from bad to worse, till it spread all over the hand. The medical superintendent blamed the probationer for it, and told her that she did not carry out his treatment, which she denies. He reported it to the Board of Guardians, and she received notice to leave. Was it right of them to dismiss her with her hand in that condition? She was out of employment for nearly 12 months, and paying for medical treatment, which amounted to a few pounds. Has she any claim?

[The case turns upon the terms of the agreement, and also as to whether the probationer obeyed, or disobeyed, the medical superintendent; and apparently the Guardians were satisfied as to her disobedience.—Ed. Hospital.]

### SLEEPING IN A PATIENT'S ROOM.

"Nurses A. and M." write: With many apologies for continuing this subject, may we thank "Medicus" through your paper for letter of 26th ult.? Personally we have received—up to now—every consideration in "private work" from medical men, but cannot help thinking while such advertisements appear as "Wanted, permanent case, skilful catheter (male or female)," there is also need for the question "Medicus" suggests, viz., "Would I let my wife, daughter, or sister undertake this?" That our training school would not permit their nurses experience in male catheter cases, and our private work has never yet called for same, we cannot feel too grateful. We firmly believe that, if nurses declined to undertake such work, both doctors and patients would have infinitely more respect for their feeling in the matter (though perhaps, at the moment, the inconvenience might cause a little temporary annoyance). To every rule in life there must occasionally occur the exception. No women have greater demands on tact and adaptability than nurses; on the other hand no women have greater need of retaining their womanliness and striving against narrow-mindedness.

"A Private Nurse" writes: I have been carefully reading the correspondence on "Sleeping in a Patient's Bedroom." I wonder what "Medicus" would do in my case. I have been with my present patient (a gentleman) for over a year and after I had been here three months my patient developed serious bladder trouble which necessitated him having the bladder washed out twice and thrice daily. The medical attendant could not possibly come so often to do this, because he lived at a considerable distance from his patient, besides having a very large scattered practice, so I was shown how to do the washing out of the bladder by the doctor. By doing this for my patient I do not feel that I have debased myself in the least, and I think that any pure, right-minded nurse would have done the same thing. Why should a nurse pass catheters, etc., in hospitals for male patients when the resident doctors are within easy call? It is a very different thing in private practice. I have been a private nurse for many years now, and my feelings are that if nurses would follow out the advice given by the nurse who wrote in the Hospital on this subject a fortnight ago, they would look upon this very necessary part of private nursing in an entirely different light to the view taken of it by "Medicus." To the pure all things are pure.

"A. E. L." writes: May I be allowed to make a few remarks upon the letter by "Medicus?" He apparently forgets that except in the larger hospitals, *i.e.* those having a resident staff, the work he so strongly condemns as "utterly" outraging a woman's feelings, has of necessity to be done by the nurses attached to the numerous small hospitals and workhouse infirmaries scattered over the country. I venture to think that there are few doctors who would consent to be called at any hour, often from a considerable distance, merely to save the feelings of a nurse, while the nurse who "refused" to do the work assigned to her would very shortly be informed that her services were no longer required. It may, perhaps, surprise "Medicus" to hear that even the larger hospitals are not all of the same mind as the two he has been connected with, as they do not scruple to send nurses on the private staff to male catheter cases if called upon to do so. This I can vouch for from personal experience. Much work that a nurse has to do is objectionable if the sex question is to be allowed to rule it, but in that case it would be unjust to look at the matter from only one point of view. Many a nurse's feelings may be fully as much "outraged" in having to stand by while a member of her own sex is being examined in the presence of a none too reverent or sympathetic group of men of all ages. It would be wiser if doctors would try to suggest practical methods by which women nurses could be relieved of some of the work they are at present obliged to do, by advocating the employment of men nurses in small hospitals, as well as for private cases, rather than by calling attention to details which are best kept in the background, and which are read by very many young nurses not to edification, as well as by a considerable number of the laity, who draw their own conclusions and make their own comments.

"Private Nurse" writes: I was very glad to see the letter from "Medicus," in which he so strongly disapproves of some of the work given to female nurses. I wish that all medical men would consider the feelings of nurses as he does. I think that a private nurse working on her own account is, to some extent, at liberty to refuse a case if she considers it unsuitable for a woman to nurse. But what of the nurse who belongs to an institution, who is sent to these objectionable cases, without having any choice in the matter? I belong to this latter class of private nurses, therefore I speak feelingly. On one occasion a surgeon sent to our institution for a nurse, and as I was the only nurse disengaged at the time, of course I was sent that evening. The patient was a bachelor of 35, living quite alone in a small flat, and he was to be operated on next morning for varicocele. The surgeon had left orders that the nurse was to take the operating table and box of dressings, etc., she was to prepare, shave, and put a compress on the patient, and have all in readiness for the operation at 10 a.m. next morning. I determined to brave the wrath of the surgeon, who was a stranger to me, so I ignored the shaving altogether, and I gave a compress to the patient to put on after his bath. I placed the necessary articles for shaving all ready, and waited to see what would happen. The three surgeons arrived, and the one who was going to assist at once proposed shaving the patient on the table The operator said the nurse had done that. I said! I had never been expected to do such a thing before, and I had not done it. The assisting surgeon and the anæsthetist at once backed me up, and the operator muttered something about supposing he ought to have got a barber to do it. I do think that male nurses ought to attend these cases, and I consider that doctors have more power to bring about this reformation than nurses themselves have. It is usually the doctor who gets the nurse for a patient. Why does he not employ more male nurses, if they are to be had? If a doctor sends to a matron for a nurse, and she has one at liberty, she can hardly refuse to send her. And if the nurse is told to go to a patient, can she refuse to do so?

[This correspondence must now cease.—Ed. Hospital.]

### Where to Go.

Midwives' Institute and Trained Nurses' Club, 12 Buckingham Street, Strand.—Friday, May 16th, address, "My Visit to South Africa," by Mrs. King Lewis. Friday, May 23rd, lecture, Dr. Tom Robinson, "Breaking Points."

### Appointments.

[No charge is made for announcements under this head, and we are always glad to receive, and publish, appointments. But it is essential that in all cases the school of training should be given.]

City of Glasgow Fever Hospital. — Miss Emily Whittle, Miss Jessie McLennan, and Miss Hannah Jardine have been appointed sisters. Miss Whittle was trained at Liverpool Royal Infirmary and has since been charge nurse under the Metropolitan Asylums Board, and sister in charge of the male wards at Stockport Union Hospital. Miss McLennan was trained at the Royal Infirmary, Inverness, where she has since been charge nurse and night sister. She has also had charge of the fever wards. Miss Jardine was trained for three years at Paisley Infirmary and Fever Hospital, and has done three years' private nursing in the North of England.

Epsom Union Workhouse.—Mrs. Alice Gardner Dibblin has been appointed superintendent nurse. She was trained at Woolwich Union Infirmary, and has since been superintendent nurse at Nantwich Union Infirmary, and assistant nurse, head nurse, and night superintendent at Woolwich Union Infirmary.

Ham Green Fever Hospital, Bristol.—Miss Fanny E. Taylor and Miss Edith J. Sime have been appointed charge nurses. Miss Taylor was trained at Salford Union Infirmary, and has since been charge nurse at Aston Union Infirmary and at Govan Parochial Hospital, Glasgow. She has lately done private nursing. Miss Sime was trained at the Royal Infirmary, Glasgow, and has since been charge nurse at Sunderland Sanatorium, Swindon Fever Hospital, and Brighton Sanatorium.

Isolation Hospital, Cheshunt.—Miss H. E. Cook has been appointed matron and Miss G. Rëyner sister. Miss Cook was trained for two years at the City Hospital, Birmingham, and has since been assistant nurse at the Brook Hospital, London; charge nurse at the Isolation Hospital, Willesden; charge nurse at the Evan Fraser Hospital, Sutton, Hull; and for two years at the South-Western Medical and Surgical Home, Putney. Miss Rëyner was trained for two years at the City Hospital, Birmingham; was assistant nurse at the Brook Hospital, London; charge nurse at the Isolation Hospital, Willesden; charge nurse at the Evan Fraser Hospital, Sutton, Hull; and for two years at the South-Western Medical and Surgical Home, Putney.

Isolation Hospital, Cippenham, Bucks. — Miss E. Griffith has been appointed matron. She was trained for three years at the Seamen's Hospital, Greenwich, where she was afterwards staff nurse for six years. She was only recently appointed charge nurse at the Isolation Hospital, Eton Union, Slough, and her new appointment is made by the committee of Eton Rural District Council.

Longton Accident Hospital, Staffordshire.—Miss Alice A. Fletcher has been appointed head nurse. She was trained at the Royal Infirmary, Manchester, and has since been sister at Fulham Infirmary.

North-Western Fever Hospital, London.—Miss E. Richards Thomas has been appointed charge nurse. She was trained at the Lambeth Infirmary for three years.

Nottingham City Isolation Hospital.—Miss Dora Berry has been appointed sister. She was trained at Sheffield General Infirmary, and has since been charge nurse under the Metropolitan Asylums Board, and staff nurse at Mansfield Accident Hospital.

Royal Infirmary, Sheffield.—Miss C. Stott has been appointed night superintendent. She was trained for three years at Birkenhead Infirmary, and was subsequently sister for twelve months. She has since been sister at St. Giles's Infirmary, Camberwell.

St. George's Home for Children, Chelsea.—Miss Ada Emily Pratt has been appointed matron. She was trained at the Victoria Hospital for Children, Chelsea, and at York County Hospital. She has since been ward sister for two years at Worcester General Infirmary, and for six months temporary matron at Budleigh Salterton Hospital.

St. Leonard's Infirmary, Shoreditch.—Miss Grace Mary Lloyd has been appointed ward sister. She was trained for three years at Sunderland Infirmary, has since been charge nurse at Lambeth Infirmary, head nurse at Lewes Union Infirmary, and superintendent nurse at Hitchin Infirmary. She holds the L.O.S. certificate.

Sheffield Workhouse Infirmary.—Mrs. Edith Davis and Miss Sarah Trainer have been appointed charge nurses. Mrs. Davis was trained for three years at Sheffield Union Infirmary, where she has since been assistant nurse. Miss Trainer was trained at Ashton-under-Lyne District Infirmary, where she has since been sister.

Skipton and District Hospital.—Miss Alice Broughton has been appointed staff nurse. She was trained at Bradford Children's Hospital, and has since been attached to the Metropolitan Convalescent Institution.

### Novelties for Nurses.

By Our Shopping Correspondent.

### FABRICS AND EMBROIDERIES.

Messrs. Jon. Harris and Sons, Limited, are holding a summer exhibition of art embroideries and needlecraft, which commenced on May 12th, and be continued throughout the season, at 25 Old Bond Street, London, W.

### THE ORIGINAL EAU DE COLOGNE.

The excellent Eau de Cologne, now known as No. 54, claims to be the pioneer brand of this now so universally-used perfume. It retains all the qualities which established the popularity of a scent which has never been surpassed. It is the one perfume which is never banished from the sick-room, where it is always acceptable for its agreeable and refreshing qualitles. No. 54 Eau de Cologne can be obtained at all chemists and perfumers, or from Messrs. Van Oppen, 38 Basinghall Street. Bottles of all sizes can be procured.

### Presentations.

Fulwood Workhouse Infirmary.—Miss Mary Back, superintendent nurse at Fulwood Workhouse Infirmary, who is about to proceed to Australia, has been presented by the Guardians of the Preston Union with a lady's dressing-case, and by the nurses in the hospital with which she has been connected for a number of years, with a handsome gold guard. Several complimentary speeches were delivered on the occasion of the ceremony.

### To Nurses.

**We invite contributions from any of our readers, and shall be glad to pay for "Notes on News from the Nursing World," or for articles describing nursing experiences, or dealing with any nursing question from an original point of view. The minimum payment for contributions is 5s., but we welcome interesting contributions of a column, or a page, in length. It may be added that notices of appointments, entertainments, presentations, and deaths are not paid for, but that we are always glad to receive them. All rejected manuscripts are returned in due course, and all payments for manuscripts used are made as early as possible after the beginning of each quarter.**

### Echoes from the Outside World.

### Movements of Royalty.

THE King has placed a beautiful stained-glass window in the private chapel at Windsor Castle, as a memorial of her late Majesty Queen Victoria. The window consists of ten lights in two tiers above the Altar. A tablet has been affixed to the oak panel work near the window, recording the memorial as follows:—"To the glory of God, and in pious memory of Victoria, Queen of Great Britain and Ireland, Empress of India, born at Kensington Palace, May 24, 1819, succeeded June 20, 1837, died at Osborne, January 22, 1901. The window above the Altar is dedicated by her devoted and sorrowing son, Edward, R.I."

The visit paid to Wales last week by the Prince and Princess of Wales afforded intense satisfaction to the Principality. They went first to Bangor, where the Prince laid the foundation-stone of the new wing of the Carnarvonshire and Anglesey Infirmary, and the Princess received purses from 60 or 70 children containing money towards defraying the expenses. A welcome announcement was made that, thanks to their Royal Highnesses, the cost of the addition to the hospital had been almost entirely met. The following day the Prince and Princess drove to Carnarvon, and the Prince was installed as Chancellor of the University of Wales in succession to His Majesty the King, whose installation to that office took place six years ago at Aberystwith. The Prince wore a robe of black satin damask with broad gold lace down the fronts, and elaborately decorated sleeves, and a velvet cap with a gold tassel. After the conference of the degree of Doctor of Laws upon the Prince, he tendered his thanks in a speech which was received with repeated cheers. In his official capacity as Chancellor of the University, the Prince conferred the degree of Doctor of Music on the Princess. Their Royal Highnesses drove back to a large public lunch at Bangor. After inspecting the Dinorwic Quarries on Saturday, when the Princess of Wales gave the signal for firing the fuse of a big blasting operation, the Royal visitors enjoyed a quiet Sunday. On the way back a visit was paid to the Royal Alexandra Hospital at Rhyl—the foundation stone of which was laid by the Queen six years ago—and the formal ceremony of opening was gone through, purses being presented to the Prince and Princess. London was reached safely on Monday evening.

### Foreign.

THE official bulletins respecting the health of the Queen of Holland continue to be entirely favourable. The physicians are said to be quite satisfied with Her Majesty's condition, and the fact that they have absented themselves from the palace the last few days for long intervals has helped to allay the apprehensions of the Dutch people. On Monday the Queen-Mother went for a drive, and as she had not been out for a week, this is regarded as evidence that the Queen's condition is very satisfactory.

One of the most terrible disasters of modern times was reported on Friday last week, namely, the total destruction of St. Pierre, the principal town in the island of Martinique, a French colony in the West Indies. The town is, or was, overshadowed by a volcanic mountain, Mont Pelée, so called because it has been entirely stripped or peeled of vegetation by the constant overflow of volcanic ash. There has been no activity in the volcano since 1851, when a slight eruption took place, and the few warning shocks and rumbles which the inhabitants experienced a few days before the disaster were not sufficient to alarm the majority, though a few of the more cautious moved to safer quarters. On Tuesday mud and lava began to flow down from the mouth of the mountain; and then, suddenly, on Thursday a mass of fire fell and burnt up the entire population of 36,000, with the exception of about 30 persons who were rescued from the sea by the cruiser *Suchet*, and later about 4,000, the population of Prêcheur. The steamer *Roddam*, although she lost several of her officers and men, was almost the only ship which escaped; but fortunately she was anchored in the outer harbour, and at the time of the catastrophe had her steam up. The captain, though seriously burnt, mounted the bridge, and amid a continuous hail of hot cinders, with portions of the ship constantly igniting, managed to move into safety. King Edward has sent £1,000; the German Emperor £500, and the United States Congress, the King of Sweden, and many other Sovereigns have forwarded contributions in money. The islands all around Martinique are doing their best to avoid deaths from starvation by sending shiploads of food, and the burial parties are cremating the bodies to avoid plague. At St. Vincent the danger is not yet over, the mountain La Soufrière being still very active and considerably upwards of a thousand lives have been lost already.

The success which attended M. Santos Dumont and his airship experiments led another Brazilian, M. Severo, to make a venture with a somewhat similar invention on Monday. His wife, who had been naturally very anxious about her husband's trial trip, had in vain endeavoured to dissuade him, and she and her son were watching the aeronaut when he set off in his machine. After rising to a height about four times as high as St. Paul's a spark from the motor ignited the balloon and M. Severo and his engineer were dashed lifeless to the ground in the presence of his horror-stricken relatives.

### Pictures at the Academy.

THE exhibits of the Royal Academy this year begin outside Burlington House. In the courtyard is an enormous gilt statue of "Edward the Black Prince," by Mr. Brock; inside the show of sculpture has little that is striking except a full length statue of Mr. Gladstone by the same artist which is to be placed in Westminster Abbey. The place of honour in the large gallery is given to the State portrait of the King, by Mr. Luke Fildes. His Majesty stands upon a daïs in front of the throne, holding the sceptre of power, clad in crimson and ermine mantle, in the attitude of conscious posing which is usually adopted in State portraits. A picture attracting much attention is that of "Queen Alexandra, her grandchildren, and dogs;" and Mr. John Charlton's "In Memoriam," a recollection of the Jubilee of 1897, is specially interesting in view of the coming Coronation. The contributions which, after the Royal pictures, are most noteworthy, are those by Mr. Sargent. He is again to the fore with his favourite groups of three Graces. This year he has two which are spoken of respectively as the "black Sargent" ("The Misses Hunter), in Room IV.; and the "white Sargent" ("The Ladies Alexandra, Mary, and Theo Acheson"), in Room II. The first represents three ladies seated back to back upon a centre ottoman, and the whole effect is sombre and rich in tone; the second pourtrays an outdoor scene, the three figures being grouped around a large terra-cotta pot containing an orange-tree laden with fruit, and the effect of dazzling whiteness and brightness is very wonderful. Mr. Sargent contributes yet another remarkable full-length portrait of "The Duchess of Portland." The President's "Storm Nymphs" is a beautiful study of nudes and still life. Sir Alma Tadema shows a monarch receiving a bevy of beautiful slaves scattering rose-leaves, entitled "Caracalla," painted in his usual finished style. Mr. John Collier has a gruesome picture, "The Plague"; Miss Lucy Kemp Welch a pathetic war incident called "Morning"; whilst Mr. Leader in "An Old Manor House," Mr. H. N. B. Davis in "Autumn Evening," and Mr. C. E. Johnson in "The Sunset of his Days," show respectively the beauties of landscape.

### The Case of Mrs. Cathcart.

AFTER five days' hearing before Mr. Justice Grantham last week, the inquiry into the state of mind of Mrs. Cathcart, who has so often been before the Courts, resulted in a unanimous verdict on the part of a special jury of 23. Their deliberations only occupied five minutes, and they decided that "Mrs. Cathcart is unable to manage herself or her affairs." On hearing this Mrs. Cathcart rose, waved her arms, and shouted, "I have not summoned a single witness, and I claim to go back to gaol, because the King's Remembrancer ——." Here, Mr. Yelverton intervened, and observed that counsel for the defence were in accord with the conclusion of the jury. Mrs. Cathcart will not be put into an asylum, but will live on her own estate under supervision, and will, of course, enjoy every comfort.

### East End Mothers' Home.

### ANNUAL MEETING.

THERE was a full attendance of visitors at the annual meeting of the East End Mothers' Home on Friday, the 9th inst. The chair was taken by Major W. Evans-Gordon, chairman of the home, who gave an account of the work and position of the institution. One hundred and ninety-eight patients, he said, had been admitted during 1901, and 329 out-patients had been attended, while 11 midwifery and 22 maternity pupils had been trained. The home was closed from July 20th to September 28th to allow the building improvements to be carried out; and the committee desired to acknowledge the care and attention received by patients during that time at the City of London Lying-in Hospital and elsewhere. King Edward's Hospital Fund had offered £200 upon condition that the money should be applied towards making during 1902 a separate labour ward on each floor, and this was being done. The £100 granted by the Fund at the end of last year was supplemented by special contributions, amounting to upwards of £120, from members of the committee, and with this help the whole of the building improvements was able to be met. There were, however, other improvements that would have to be made, and he strongly appealed for funds "to render this lying-in home one of the most efficient in London."

The Bishop of Stepney, in moving the adoption of the report and accounts, said that if there was one sore point in the lives of the poor, it was the condition in which the children were born into the world. More than once he had had to clear the room where a woman lay ill of a crowd who were celebrating the event after the manner of Mrs. Harris and Mrs. Gamp. With the well-to-do such an event in a woman's life was regarded almost with reverence, and the contrast presented by the lives of the poor was hardly conceivable. He could not help feeling that the way in which the mother herself regarded the birth of the child must govern the whole of their future relationship: if it came as an additional burden, or a nuisance, it would remain so; it should, on the contrary, be a refreshment to the parents' love, and any home that helped to give the mother a taste of peace, kindness, sympathy, and beauty during her hour of trial was one to be commended. He hoped that God's blessing would rest on all who gave their work, as nurses or in any other way.

The motion was seconded by the Rev. H. Woollcombe, head of the Oxford House, who said that he had had personal experience of the good done by the home. Anything that brought a trained nurse to the child who came into the world handicapped, in the way described by the previous speaker, in its earliest years was of immense value. Sickness often had an extraordinary softening effect, and it was a great opportunity for giving sympathy and help to the mothers who came into the home as patients.

The report and accounts were unanimously adopted, and after a collection had been made, the visitors were entertained to tea and coffee by Miss Alice Blomfield, the new matron, and the nurses. The opportunity was taken of visiting the wards and inspecting the building improvements referred to by the chairman.

### TRAVEL NOTES AND QUERIES.

SOMEWHERE NEAR MUNICH (M. E. W.).—How would Partenkirschen suit you? it is an exquisite spot, 60 miles from Munich and one mile from Garmisch. Either of these places is charming and would not be expensive; at Partenkirschen try Pension Schweizerhaus, they will, I think, take you from five marks a day. At Garmisch, Pension Hohenleitner, about the same terms. Then there is Salzburg, in the heart of beautiful country. Write there to Pension Yung, I do not know their terms, but in Salzburg I should be inclined to go to a modest hotel, such as Hotel Krebs, on the Mirabell-Platz, and make inquiries. For the Rhine, Coblentz is a splendid situation, because you command the Moselle country as well—see my papers last year for July 13th and July 20th. At Coblentz try the Hotel de Trêves in the Clemens-Platz, very moderate. As to hearing only German spoken, the Continent is so overrun by the English and the Germans learn our language so very quickly that almost the only way now is to talk a great deal with the servants, small shopkeepers in retired places, all the employees you meet in your travels, etc. . . . For the Black Forest you will find Hornberg a good centre; there are no pensions there, but a very reasonable and thoroughly German hotel, the Post, or the Bär equally good, both about five marks per day.

### For Reading to the Sick.

### WHITSUNTIDE.

ONWARD the Solemn Feast-day rolled,
Upon its seven-fold circle borne;
The mystic week of weeks, that told
The coming of that blessed morn.

It comes, the third Hour of the day,
While thunder shakes the world's wide dome,
And, as the blest Apostles pray,
Heralds aloud that God doth come.

The Holy Spirit breathes abroad,
And while their freshened hearts rejoice,
They speak the mighty Works of God
With varying tongue, but one true Voice.

*From the Latin.*

"I believe in the Holy Ghost, the Lord, and the Life-giver." The Christian doctrine of the Holy Spirit covers a wide area, and has far-reaching results. . . . In confessing the Holy Spirit to be the Giver of Life, or the Maker-alive, we mean that all life, whether in nature or in man, is His presence and gift. "It is the Spirit that quickeneth." "The Spirit is life." "Wherever the Spirit is," says St. Ambrose, "there is also life; and wherever life is, there is also the Holy Spirit." The very laws of nature are the Spirit's methods. The Creator and Sustainer of the natural world regards His work as a whole, as one vast body: and intelligent Christians believe that the breath or life of that body proceeds from the Person of the Life-Giver—in fact, that this life is His presence and His gift. The writer of the one hundred and fourth psalm, the great hymn of praise for God's goodness as manifested in the natural world, after looking round upon the works of creation and seeing in them the sustaining presence of God, exclaims:

"Thou takest away their breath, they die,
And return to their dust.
Thou sendeth forth thy Spirit, they are created;
And thou renewest the face of the ground."

The whole psalm follows the order of creation given in Genesis i., where the account is prefaced by the statement that "The Spirit of God," as the great vitalising principle, "moved (or, was brooding) upon the face of the waters."

As the Life-Giver, the office of the Spirit is to bring us into union with Jesus Christ, who is the Life. In possessing the Spirit, the Christian possesses Christ, and so can say, "I can do all things in Him that strengtheneth me." It is the presence of the Holy Spirit within the soul, which adequately empowers the will to act aright in face of temptation, difficulty, or danger.—*Rev. V. Staley.*

Ah! princely Spirit, thine the skill,
The heart to melt, to brace the will,
The soul to beautify:
A Christ-like life in all its strength,
In all its sweetness—this at length
Must win the victory.

Bestow thy gifts, thy grace dispense,
O guard the avenues of sense,
And keep our garments white;
Enrich our poverty, and make
Our weakness strength, for Jesu's sake,
And all our darkness light.

*Anon.*

### Notes and Queries.

**The Editor is always willing to answer in this column, without any fee, all reasonable questions, as soon as possible.**
**But the following rules must be carefully observed :—**
**1. Every communication must be accompanied by the name and address of the writer.**
**2. The question must always bear upon nursing, directly or indirectly.**
**If an answer is required by letter a fee of half-a-crown must be enclosed with the note containing the inquiry, and we cannot undertake to forward letters addressed to correspondents making inquiries. It is therefore requested that our readers will not enclose either a stamp or a stamped envelope.**

*Pelvis. Skull.*

(56) Will you kindly tell me where I can obtain a female pelvis and fœtal skull, and the average cost ?—*Norah.*

From the surgical instrument makers, or through your local chemist, or by advertisement. Nurses sometimes try to dispose of these things in our columns.

Will you kindly tell me where I can obtain a pelvis and fœtal head for hire, or where I can buy them cheaply ?—*Sister and M. T.*

See reply to *Norah.*

*Spectacles.*

(57) I have been told that there is some institution in London where needy people can be supplied with spectacles. Can you give me the address ?—*A. E. B.*

The London Spectacle Mission Society, 197 Sutherland Avenue, W.

*Greece.*

(58) Can you give me any information regarding the English Hospital at Greece ? To whom should I apply, what would be the cost, and what clothes should be taken ?—*Erin go Bragh.*

Do you mean St. Sophia's Children's Hospital, Athens ? If so, apply to the Matron, enclosing stamps for reply.

*South Africa.*

(59) Could you give me any information how to get into a hospital in South Africa ?—*A. L. H.*

If you are a trained nurse, the Hon. Secretary, the Victoria Nurses' Institute, Capetown, would be able to advise you.

*" Gray's Anatomy."*

(60) Can you me where I can get a second-hand copy of " Gray's Anatomy " cheap ? It is an expensive book.—*F. G.*

Possibly some of our correspondents will be glad to dispose of theirs. The second-hand booksellers in the neighbourhood of any of the large medical schools often have old editions of " Gray's " for sale.

*Hospital Training.*

(61) I should be glad if you could tell me where I could get one year's training in a children's hospital free ? I am in a school, and am anxious to learn a little nursing, but I cannot afford to pay a premium.—*D. J.*

See " The Nursing Profession : How and Where to Train." Why not enter for a three years' certificate ? It will be exceedingly difficult to get one year's training free, whilst there are many good schools which pay a salary from the first year for the full course of three years.

I see that there are several advertisements in The Hospital which direct replies to be sent to The Hospital Office, and I thought that perhaps you would be kind enough to let me know if there is any Women's and Children's Hospital where a probationer is wanted as soon as possible after July ?—*W. C.*

See the rules given to correspondents at the head of this column. See, for list of training schools for probationers, " The Nursing Profession : How and Where to Train."

What is the best way of getting a village girl of 21 trained as nurse ? She cannot afford to pay a premium, and the institutions to which I have applied prefer educated women.—*W. S.*

If the girl has the qualities for making a good nurse, there are plenty of institutions which will train her when she is 23. See " The Nursing Profession : How and Where to Train."

Could you tell me if it is possible for a girl, not very well educated, to obtain training as a hospital nurse ?—*N. S.*

If the girl has a good elementary education she should apply to some of the Poor Law Infirmaries for training. She will find a list in " The Nursing Profession : How and Where to Train."

I have studied " The Nursing Profession : How and Where to Train," and I think that either the Bristol General Hospital or the Lincoln County Hospital would be most suitable. Will you kindly tell me if these are first rate training schools ?—*Naomi.*

Yes.

I am 17. Will you kindly tell me how I can prepare to become a nurse. Would house and book-keeping be of any service ?—*N. F.*

If ever you are fortunate enough to become sister or matron, it would be absolutely essential for you to understand book-keeping, house-keeping, the care of linen, high-class cookery, etc.

I am anxious to get into a small hospital of about 50 beds for two years' training. I have a certificate for one year's training and was accepted by a large hospital in London, but I broke down after the first three months. Can you recommend a school ?—*E. M.*

You will have no difficulty in finding a suitable training school amongst the smaller hospitals. " The Nursing Profession : How and Where to Train," gives a full list. Try and negotiate a three years' continuous certificate if possible.

Is it possible for a nurse who has had three months' maternity training only to get an appointment as assistant matron or some other post in order to gain experience in medical nursing, as she is too old to enter as probationer ?—*I. W.*

It is not probable that she could get a position as matron, but there may be other posts in private homes or small hospitals which a capable woman might obtain, and where she could gain some knowledge of medical nursing. You had better advertise.

I am anxious to get a few months' training in a hospital for chest diseases with a view to obtaining a post in an open-air sanatorium. Will you kindly tell me of any hospital where paying probationers are received ?—*E. S.*

You could probably acquire the modicum of training which you require at an open-air sanatorium ; you would be wise however to take a full course of training if you intend earning your living as a nurse. The Mount Vernon Hospital for Consumption, Hampstead, gives a two years' certificate and pays a salary from the first.

*Maternity.*

(62) Would it be advisable for me to take the L.O.S. as well as the certificate from the Rotunda Hospital ? Ought I to take a three years' certificate in general nursing also ?—*Nurse D. H.*

In order to become a first-rate maternity nurse you ought to be trained in general nursing as well as in maternity. The certificate which you already hold, together with one year's certificate in general nursing and two years' in gynæcology from one of the special women's hospitals should be sufficient for all practical purposes.

Can you tell me if there is an institution at Rouen from which an English maternity nurse could be obtained ? Also, can you give me the name of a nursing home in Paris which takes maternity cases ? Are such cases received at the Hertford Hospital, Paris ?—*Nurse.*

We have not the name of any nursing association supplying English nurses at Rouen, but it is possible that the English chaplain there may know of one privately. The secretary of the Hertford Hospital, Rue de Villiers-Levallois-Perret, Paris, will give you the information you require.

I am a monthly nurse and have plenty of cases, but I should like to get a certificate from the L.O.S. Can I do this by reading up under a doctor, or must I go into hospital again. I cannot afford either the time or the money to do this.—*R. D. W.*

Yes, a medical man can prepare you for the L.O.S. examination. Write to the secretary of the society, 20 Hanover Square, London, W., for syllabus.

Is there a suitable institution in Portsmouth or Southsea where I could train as midwife ?—*A. M. T.*

There is no public institution for training midwives at either of these places, but there are sure to be private homes and teachers. Write and ask the secretary, the Midwives' Society, 12 Buckingham Street, Strand, W.C., to recommend one.

Could you tell me where I could get a cheap course of training in maternity ?—*Nurse G. K.*

The British Lying-in Hospital, Endell Street, St. Giles, W.C. ; the City of London Lying-in Hospital, City Road, E.C. ; and the East End Mothers' Home, 394 and 396 Commercial Road, E., are all good and cheap training schools in maternity nursing.

*Home.*

(63) Can you tell me of a permanent home for a strong woman of 34 ? She had a stroke of paralysis as an infant and is unmanageable at home. Her mother could afford to pay 10s. a week for her.—*Nurse S.*

This appears a case which might be dealt with in a private home ; possibly the Secretary of the National Association for Promoting the Welfare of the Feeble-minded, 53 Victoria Street, S.W., would be able to recommend one.

**Standard Nursing Manuals.**

" The Nursing Profession : How and Where to Train." 2s. net ; post free 2s. 4d.
" Art of Massage." (Second Edition.) 6s.
" Elementary Physiology for Nurses." 2s.
" Elementary Anatomy and Surgery for Nurses." 2s. 6d.
" Practical Handbook of Midwifery." 6s.
" Surgical Ward Work and Nursing." Revised Edition. 3s. 6d. net ; post free 3s. 10d.
" Mental Nursing." 1s.
" Art of Feeding the Invalid." 1s. 6d.
All these are published by the Scientific Press, Ltd., and may be obtained through any bookseller or direct from the publisher, 28 and 29 Southampton Street, London, W.C.

## Figures and Tables

**Fig. 42. f1:**
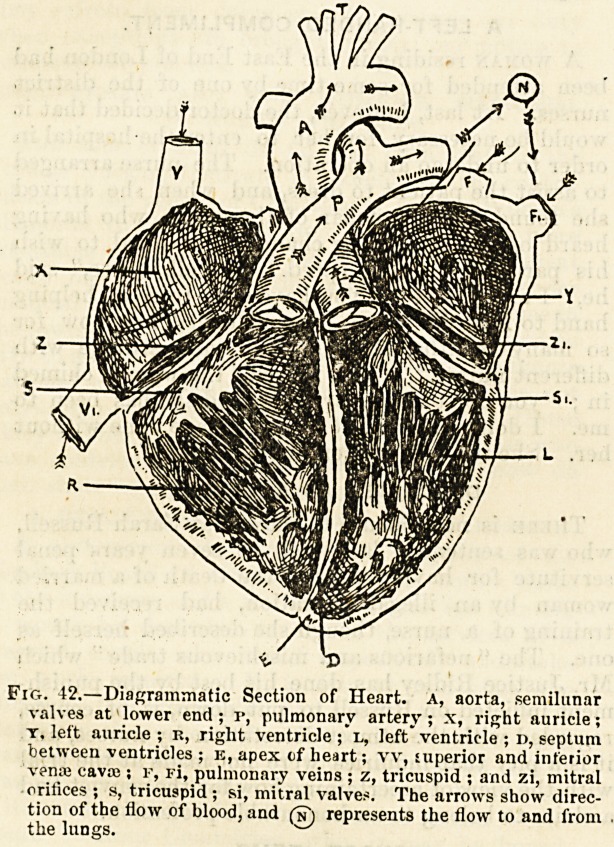


**Fig. 43. f2:**